# Maternal immune activation induces autism-like changes in behavior, neuroinflammatory profile and gut microbiota in mouse offspring of both sexes

**DOI:** 10.1038/s41398-022-02149-9

**Published:** 2022-09-14

**Authors:** Anna Maria Tartaglione, Annacandida Villani, Maria Antonietta Ajmone-Cat, Luisa Minghetti, Laura Ricceri, Valerio Pazienza, Roberta De Simone, Gemma Calamandrei

**Affiliations:** 1 Centre for Behavioral Sciences and Mental Health, Italian National Institute of Health (ISS), Rome, Italy; 2 Gastroenterology Unit IRCCS “Casa Sollievo della Sofferenza”, Hospital San Giovanni Rotondo, Foggia, Italy; 3 National Centre for Drug Research and Evaluation, Italian National Institute of Health (ISS), Rome, Italy; 4 Research Coordination and Support Service, Italian National Institute of Health (ISS), Rome, Italy

**Keywords:** Autism spectrum disorders, Molecular neuroscience

## Abstract

Autism Spectrum Disorder (ASD) is a sex-biased neurodevelopmental disorder with a male to female prevalence of 4:1, characterized by persistent deficits in social communication and interaction and restricted-repetitive patterns of behavior, interests or activities. Microbiota alterations as well as signs of neuroinflammation have been also reported in ASD. The involvement of immune dysregulation in ASD is further supported by evidence suggesting that maternal immune activation (MIA), especially during early pregnancy, may be a risk factor for ASD. The present study was aimed at characterizing the effects of MIA on behavior, gut microbiota and neuroinflammation in the mouse offspring also considering the impact of MIA in the two sexes. MIA offspring exhibited significant ASD-like behavioral alterations (i.e., deficits in sociability and sensorimotor gating, perseverative behaviors). The analysis of microbiota revealed changes in specific microbial taxa that recapitulated those seen in ASD children. In addition, molecular analyses indicated sex-related differences in the neuroinflammatory responses triggered by MIA, with a more prominent effect in the cerebellum. Our data suggest that both sexes should be included in the experimental designs of preclinical studies in order to identify those mechanisms that confer different vulnerability to ASD to males and females.

## Introduction

ASD is a sex-biased neurodevelopmental disorder characterized by persistent deficits in social communication and interaction and restricted-repetitive patterns of behavior, interests or activities [1]. Growing evidence indicates the neuroimmune system as a key player in ASD pathogenesis [2, 3], as evidenced by microglial and astrocytic activation, and increased expression of pro-inflammatory cytokines in the cerebrospinal fluid (CSF) and brain of children with ASD [4–10].

The involvement of immune dysregulation in ASD is supported by epidemiological studies suggesting that maternal infection early in gestation is a risk factor [11, 12]. The role of maternal infection and the consequent maternal immune activation (MIA) in ASD is further corroborated by evidence in mouse models, where MIA induces in offspring long-lasting changes in behavior recapitulating core symptoms of ASD [12]. However, the exact mechanisms through which prenatal immune activation affects early brain and behavior development are not yet fully understood.

Recent evidence points to gut microbiota as a critical determinant of ASD-like behavioral abnormalities [13]. Children with ASD are 4 times more likely to suffer than neurotypical children from gastrointestinal (GI) disorders whose severity is related to that of their behavioral symptoms [14, 15]. Moreover, GI symptoms are associated with changes in the gut microbiota of ASD individuals [13–15]. Not least, microbiota is crucial for the programming and presentation of normal social behavior in mice [16, 17]. Seminal findings on germ-free (GF) mice showed that host microbiota is also an essential environmental factor shaping microglia maturation and activation, both in steady state and pathology [18, 19]. Microglia, the resident immune cells of the central nervous system (CNS), have a pivotal role in many neurodevelopmental processes such as synaptic pruning, maturation of brain circuitry, and immunosurveillance [20, 21]. This population of neuroimmune cells is of particular interest in sex-biased neurodevelopmental disorders such as ASD because during physiological development microglia undergo a sex-dependent maturation process, which is delayed in males relative to females [22]. Interestingly, the effects of microbiota depletion on microglial function in offspring are also sex- and age-related with a greater impact in males during prenatal development while in females at adulthood [19]. Unfortunately, sex differences in microglia and microbiota in a context of early adversity such as maternal infection remain underexplored. Indeed, while most studies on MIA did not report the sex of animals used, others have only analyzed males or males and females together [23]. Considering the complex interaction between the CNS, immune system and gut microbiota as well as the strong sex bias in ASD, our study was designed as to investigate sex-related differences in the response of mouse offspring to a single dose of viral mimetic polyinosinic:polycytidylic (Poly I:C), administered to the mother on gestational day (GD) 12.5. This is a time period mirroring the first trimester of pregnancy in humans when maternal viral infection has been associated with increased incidence of ASD [24, 25]. Firstly, we explored the effects of prenatal Poly I:C on ASD-like behavioral phenotype in young/adult male and female mice. We then investigated the consequence of the prenatal immune challenge on (i) neuroinflammatory response in hippocampus and cerebellum, two brain regions widely related to behavioral alterations typical of ASD, and (ii) gut microbiota composition at various taxonomic levels.

## Material and methods

### Animals

Experiments were carried out in accordance with the EU and Italian legislation (2010/63/EU, Dl 26/2014) and approved by the Animal Welfare Committee of the Italian National Health Institute (Istituto Superiore di Sanità, ISS) and by the Italian Ministry of Health.

Six-week C57BL6/J mice (Jackson, Bar Harbour, ME, USA) were housed under standard conditions (temperature 21 ± 1 °C and relative humidity 60 ± 10%), under a 12:12 reverse light cycle (lights on at 6:00 P.M.). After mating (2 females: 1 male) females were checked twice a day for the presence of the vaginal plug (gestational day, GD, 0).

At GD 12.5 pregnant mice received a single injection of Poly I:C [(potassium salt; Sigma-Aldrich, #P9582), (20 mg/kg, i.p.)] or vehicle (Veh, 0.9% NaCl). All pups remained with their mother (*n* = 12 litters for each treatment group) until post-natal day (pnd) 28 when they were housed with same-sex littermates (2–3 mice per cage). For each analysis only one pup for sex from each litter was employed.

### Behavioral testing

One male and one female randomly selected from each litter (12 males and 12 females for each treatment group) underwent behavioral testing between 9.00 AM and 2.00 PM. i.e., during the dark phase of circadian cycle. Sequence of behavioral testing was: Open-Field (pnd 49), Elevated-plus maze (pnd 56), Three-chamber social test (pnd 63), Spontaneous alternation in T-maze (pnd 77), Acoustic Startle Response and Prepulse inhibition (pnd 120).

#### Open-Field test (OF)

OF test assesses the locomotor activity while exploring a novel environment. The OF apparatus consisted of a black Plexiglas box (40 × 40 × 40 cm). Each mouse was placed in one corner of the apparatus and spontaneous locomotor activity of the animals video-recorded for 10 min. Distance traveled and mean velocity were analyzed using ANY-Maze software (Stoelting Europe, Dublin, Ireland).

#### Elevated Plus Maze (EPM)

To assess the anxiety-like behavior, mice were tested in the EPM based on the conflict between the exploration of new areas and avoidance of unsafe areas. The EPM was a Plexiglas cross-shaped maze, 60 cm high above the floor, consisting of two open and two closed arms. Each mouse was placed in the center of the maze facing an open arm and allowed to explore the maze for 5 min. Frequencies of total, open and closed entries (all four paws into an arm) and time spent in each arm were analyzed by a trained investigator blinded to experimental group using The Observer XT-15 software (Noldus, Wageningen, The Netherlands).

#### Three-chamber social test

The three-chamber apparatus was a Plexiglas box (60 × 40 cm) divided into three chambers connected by doorways as described previously [26]. The subject mouse acclimated to the empty apparatus for 10 min before the sociability test. The subject was then confined to the center chamber. An object enclosed in an inverted wire cup was introduced into one of the side chambers while an unfamiliar, age- and sex-matched mouse was placed under an identical wire cup in the other side chamber. The subject was then allowed to access to all three chambers for 10 min. Side chamber location of the object and the social stimulus were counterbalanced across subjects. The time spent in each chamber and the time spent sniffing each cup were recorded and analyzed by a trained investigator blinded to experimental group using The Observer XT-15 software (Noldus, Wageningen, The Netherlands). A sociability index (SI) was calculated as follows: (time sniffing social stimulus−time sniffing object)/(time sniffing social stimulus+ time sniffing object).

#### Spontaneous alternation in T-maze

To assess perseverative behaviors, mice were tested in the T-maze test, based on the natural tendency of rodents to alternate their choice of goal arm. The apparatus was a T-shaped maze consisting of three equally sized arms (50 × 16 cm). Six sessions, consisting of two-choice trials, were performed on three consecutive days (two sessions per day). Mice were placed at the base of the T (starting arm) and allowed to explore the apparatus, until it entered one of the two arms or for a maximum of 2 min. Immediately after the mouse made the first arm choice, it was moved to the starting arm, to perform the second-choice trial. If the mouse entered the arm opposite to the previously chosen one, an alternation was scored. The percentage of alternations was computed as the number of alternations divided by the number of completed sessions × 100. Data were scored manually by a trained investigator blinded to experimental group.

#### Acoustic Startle Response (ASR) and Prepulse Inhibition (PPI)

Sensorimotor gating abilities of mice were assessed through the PPI paradigm that evaluates the reduction in the acoustic startle response (ASR) upon presentation with a weak prepulse stimulus. The apparatus (Med Associates Inc. St Albans, VT,US) was a Plexiglas rectangular box (9 × 7 cm), positioned on a platform with a transducer amplifier (PHM-250-60) in a sound-attenuating chamber (ENV-018S), and an acoustic stimulator (ANL-925) and it was controlled by a dedicated software (SOF-815).During habituation (day 1), each mouse was left undisturbed in the apparatus for 5 min. On the following day (day 2), mice were exposed to the background noise (62 dB) for 5 min and subsequently subjected to three blocks of trials. The first and third blocks consisted of 10 trials (presentation of 10 pulses of 120 dB) interspaced by an average inter-trial interval of 15 s. The second block consisted of 56 trials, comprising four different types of trial (prepulse alone, prepulse plus pulse, startle alone, and no stimulation) in a pseudorandomized order. The intensity of the prepulse was 74, 78, 82, or 84 dB. Following the prepulse, a startle stimulus (40-ms long white noise, 120 dB of intensity) was presented. The galvanic response was measured 65 ms after the onset of the startle. In the first and the third blocks the parameter measured was the ASR. The second block was used to measure the PPI as follows: [(A − B)/A × 100], where in A is the Galvanic reflex registered after the startle stimulus alone, and B is the reflex registered in response to the startle in prepulse plus pulse trials.

### Quantification of gene expression using RT-PCR on hippocampus and cerebellum

Behaviorally naïve offspring of both sexes were sacrificed at pnd 28 and 120 and samples of hippocampus and cerebellum collected and stored at −80 °C. For gene expression experiments, RNA was extracted using Trizol reagent. The quality and concentration was measured at Nanodrop. cDNA was reverse transcribed from 1 µg of RNA using the High-Capacity cDNA Reverse Transcription Kit (Applied Biosystems, Thermo Fisher Scientific). Real-time PCR was performed on the reverse transcription products with TaqMan master mix and TaqMan™Gene Expression Assays (Applied Biosystems, Thermo Fisher Scientific). As housekeeping gene, we utilized hypoxanthine guanine phosphoribosyl transferase (HPRT). Annealing temperature was 60 °C for all the primer pairs listed. All samples were run in duplicate, and each PCR well contained 20 μl as a final volume of reaction, including 2 μl of complementary DNA corresponding to ~25 ng total RNA, 750 nM of each primer, and 10 μl PCR master mix. Thermal cycling conditions were as follows: 1 cycle at 95 °C for 10 min, 40 cycles at 95 °C for 15 s, and 60 °C for 1 min. The relative expression level of each mRNA was calculated using the 2^−ΔΔCt^ method, normalized to HPRT and relative to Veh mice.

### 16S rRNA sequencing and analysis on fecal samples

Fecal samples were freshly collected from each mouse cage at pnd 28 and 120 and stored at −80 °C within 1 h. Microbial DNA was extracted from fecal samples using the Qiagen stool kit (Qiagen, Milan, Italy) as described by the manufacturer and then the V3–V4 hypervariable regions of the bacterial 16 S ribosomal RNA was amplified by PCR using barcoded universal primers [selected from [27]] here reported: forward primer: 5'-TCGTCGGCAGCGTCAGATGTGTATAAGAGACAGCCTACGGGNGGCWGCAG, reverse primer: 5′-GTCTCGTGGGCTCGGAGATGTGTATAAGAGACAGGACTACHVGGGTATCTAATCC. The PCR products were purified by means of Agencourt AMPure XP beads (Beckman Coulter, Milan, Italy), quantified and then subjected to a further PCR to attach dual Illumina indices (Nextera XT Index Kit, Illumina Inc., San Diego, CA, USA) necessary for multiplexing and equimolar ratios of amplicons from individual samples were pooled before sequencing on the Illumina platform using high throughput screening Illumina MiSeq instrument. Sequence data generated as FASTQ files, deposited in the Arrayexpress repository under accession code E-MTAB-11150, were analyzed using the 16S Metagenomics GAIA 2.0 software as already described [28]. Read pairs were quality-controlled (i.e., trimming, clipping and adapter removal) based on FastQC and BBDuk and mapped with BWA-MEM against the 16 S databases ((GAIA based on NCBI), to obtain the taxonomic profile of each sample).

### Statistical analysis

The sample size has been calculated using G*Power 3.1 software setting test power 1–β = 0.8 and effect size *f* = 0.5 (large effect size) using analysis of variance (ANOVA) and α = 0.05/number of useful comparisons. All data (behavior, neuroinflammation, microbiota) were analyzed by two-way ANOVA with prenatal administration (Veh or Poly I:C) and sex as between-factors followed by post-hoc Tukey’s test on significant interaction effects. ANOVA with repeated measures was also performed with zones/arms/chamber/stimulus intensity as within factors. The arcsin transformation was applied to microbiota data before performing ANOVA. Potential outliers were detected by Grubbs’ method available in the GraphPad software. Pearson correlations were applied to associate the bacterial taxa, with neuroinflammatory markers, as well as with behavioral parameters (measured in littermates) considering all animals, regardless of their prenatal treatment. Statistical analyses were performed with GraphPad Prism 8.3.1.

## Results

### MIA induces ASD-like behavioral alterations in adult offspring of both sexes

**Fig. 1:**
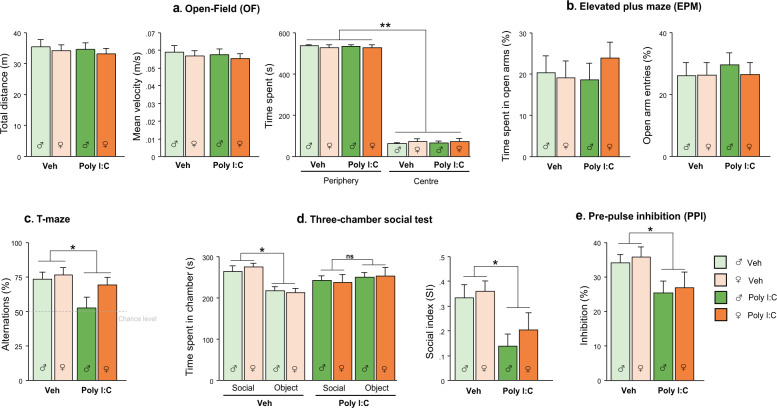
Behavioral effects of MIA in juvenile/adult offspring. MiA offspring of both sexes did not show alterations in locomotor activity (**a**) and anxiety-like behavior (**a**, **b**). MIA significantly affected spontaneous alternation (**c**), social discrimination (**d**) and sensorimotor gating (**e**) in both males and females (*n* = 12 M/12 F per group). Data are shown as means ± S.E.M. Two-way ANOVA with post-hoc Tukey’s test, *p < 0.05; **p < 0.01; ns = *p* > 0.05.

No significant differences were found in locomotor activity and anxiety-like behavior measured in OF and EPM, between Poly I:C and Veh mice (Fig. 1a, b). However, Poly I:C offspring of both sexes showed a lower percentage of spontaneous alternation in T-maze compared to Veh offspring [main effect of treatment: F(1,44) = 5.627, *p* < 0.05], with males more impaired as shown by the below chance level performance [95% confidence intervals (CI), Veh females: 64.91, 87,86; Veh males: 62.14, 85.09; Poly I:C females: 58.54, 80,39; Poly I:C males: 35.98, 69.57], Fig. 1c.

Poly I:C offspring, regardless of sex, exhibited decreased preference for the social stimulus in the three-chamber test, as they spent less time exploring the social chamber [treatment*chamber: F(1,44) = 6.517, *p* < 0.05, *p* < 0.01 after post-hoc comparisons] and sniffing the social stimulus as indicated by social index (SI), [main effect of treatment: F(1,44) = 10.641, *p* < 0.01] compared to Veh mice (Fig. 1d). Specifically, Poly I:C offspring were more interested in the object compared to Veh mice [treatment*stimulus: F(1,44) = 8.577, *p* < 0.01; sniffing object: *p* < 0.05 Poly I:C *vs* Veh (sniffing duration, Veh: social = 62.838 ± 2.934, object = 30.003 ± 1.678; Poly I:C: social = 58.069 ± 3.681, object = 41.027 ± 3.157)].

While the analysis of ASR on the pulse-alone trials revealed a comparable response to a 120 dB startle stimulus between Poly I:C and Veh offspring, the analysis of percentage of PPI showed a main effect of Poly I:C treatment [F(1,44) = 6.768, *p* < 0.05] with MIA offspring displaying reduced PPI compared to Veh offspring (Fig. 1e).

### MIA differentially affects the neuroinflammatory profile in the hippocampus and cerebellum of male and female offspring

We analyzed the expression of genes involved in homeostatic and immune functions of microglia and markers of neuroinflammation (the pro-inflammatory cytokines TNF-α, IL-6 and IL-1β, anti-inflammatory cytokine TGF-β, the inflammatory/oxidative stress-related enzymes iNOS and ARG-1, the triggering receptor expressed on myeloid cells (TREM2), the microglia-specific marker TMEM119, the microglia/macrophage activation marker CD68, the astrocytic activation marker GFAP, the neurotrophin BDNF) in hippocampus and cerebellum at juvenile and adult stage. Notably, accumulating evidence point to a cerebellar contribution in higher functions such as emotional, social and cognitive processing [29, 30].

**Fig. 2:**
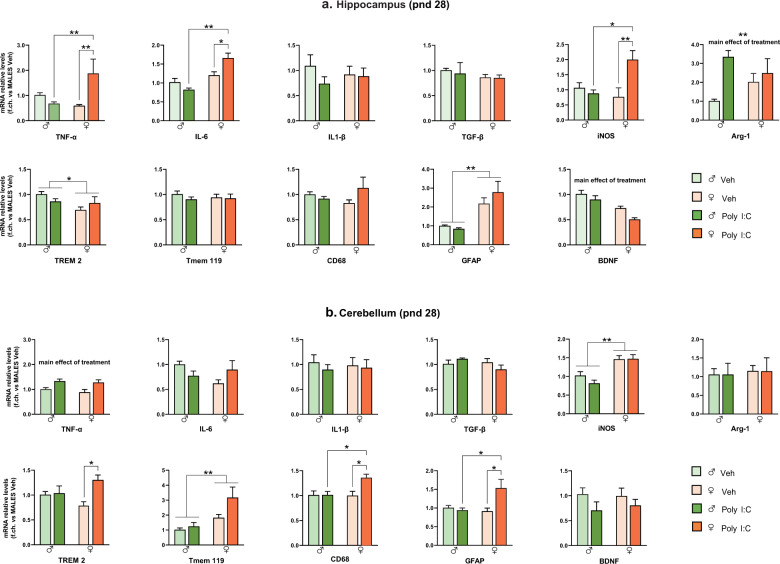
MIA effect on neuroinflammatory profile of offspring at pnd 28. mRNA levels in hippocampus (**a**) and cerebellum (**b**) were evaluated by RT-PCR using the 2^−ΔΔCt^ method, normalized to the housekeeping gene β-actin, and relative to Veh males (*n* = 6 M/6 F per group). Data are expressed as means ± S.E.M. Two-way ANOVA with post-hoc Tukey’s test, *p < 0.05; **p < 0.01.

Basal expression of these genes, as well as their regulation by prenatal Poly I:C exposure, were largely sex-, age- and brain region-specific. In the hippocampus at pnd 28 (Fig. 2a), Poly I:C induced a strong increase of TNF-α [treatment*sex: F(1,17) = 13.4, *p* < 0.05, *p* < 0.01 vs Veh females and Poly I:C males], IL-6 [treatment*sex: F(1,16) = 11.66, *p* < 0.01, *p* < 0.05 vs Veh females and *p* < 0.01 *vs* Poly I:C males] and iNOS [treatment*sex: F(1.19) = 8.495, *p* < 0.01, *p* < 0.01 vs Veh females and *p* < 0.05 *vs* Poly I:C males] only in females. In addition, Poly I:C decreased BDNF and increased Arg-1 in the offspring of both sexes [main effect of treatment F(1,18) = 9.225, *p* < 0.01 for BDNF and F(1,17) = 6.805, *p* < 0.05 for Arg-1]. The other mRNAs analyzed (IL-1β, TGF-β, TREM2, Tmem119, CD68, and GFAP) were not altered by prenatal Poly I:C administration. However, expression of some of these genes were sex-specific: females, regardless of prenatal treatment, expressed lower TREM2 and higher GFAP levels compared to males [main effect of sex: F(1,20) = 4.870, *p* < 0.05, F(1,18) = 9.225, *p* < 0.01, respectively].

In the cerebellum (pnd 28, Fig. 2b), Poly I:C increased the expression of TREM2 [treatment*sex: F(1,18) = 5.258, *p* < 0.05, *p* < 0.05 *vs* Veh females], CD68 [treatment*sex: F(1,18) = 5.277, *p* < 0.05, *p* < 0.05 *vs* Veh females and Poly I:C males] and GFAP [treatment*sex: F(1,17) = 7.375, *p* < 0.05, *p* < 0.05 *vs* Veh females and Poly I:C males] only in females. Moreover, Poly I:C upregulated TNF−α mRNA levels [main effect of treatment F(1,16) = 13.14, *p* = 0.002] in offspring of both sexes. Females, regardless of prenatal treatment, expressed higher iNOS and Tmem119 levels compared to males [main effect of sex: F(1,17) = 30.85, *p* < 0.001; F(1,15) = 11, *p* < 0.01, respectively]. These genes, as well as IL-6, IL-1β, TGF-β, Arg-1, and BDNF, were not affected by prenatal Poly I:C exposure.

**Fig. 3:**
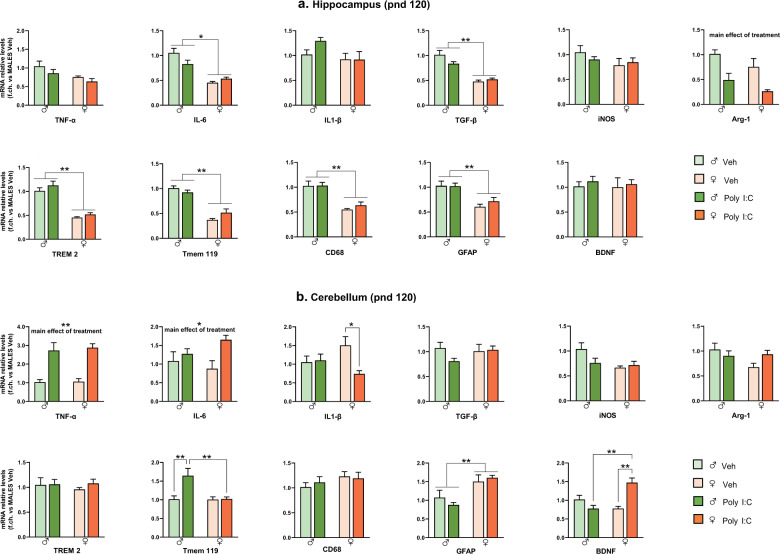
MIA effect on neuroinflammatory profile of PND 120 offspring. mRNA levels in hippocampus (**a**) and cerebellum (**b**) were evaluated by RT-PCR using the 2^−ΔΔCt^ method, normalized to the housekeeping gene β-actin, and relative to Veh males (*n* = 6 M/6 F per group). Data are expressed as means ± S.E.M. Two-way ANOVA with post-hoc Tukey’s test, *p < 0.05; **p < 0.01.

At pnd 120, in the hippocampus (Fig. 3a), Poly I:C induced Arg-1 downregulation of in both males and females [main effect of treatment: F (1,16) = 19.55, *p* < 0.001], while it did not affect the expression of the other genes. Notably, most of these genes were expressed at lower levels in females (regardless of treatment) as compared to males [main effect of sex: F(1,19) = 6.464, *p* < 0.05 for TNF-α; F(1,20) = 40.23, *p* < 0.001 for IL-6; F(1,19) = 76.43, *p* < 0.001 for TGF-β; F(1,20) = 98.53, *p* < 0.001 for TREM2; F(1,19) = 93.97, *p* < 0.001 for Tmem119; F(1,19) = 37, *p* < 0.001 for CD68; F(1,20) = 23.43, *p* < 0.001 for GFAP]. In the cerebellum (Fig. 3b), Poly I:C upregulated TNF-α and IL-6 in offspring of both sexes [main effect of treatment F(1,16) = 33.75, *p* < 0.001 for TNF-α; F(1,16) = 7.628, *p* < 0.05 for IL-6]. In Poly I:C females, the increase of TNF-α and IL-6 was also accompanied by BDNF increase [treatment*sex: F(1,18) = 22.52 *p* < 0.001, *p* < 0.01 *vs* Poly I:C males and Veh females] and IL-1β decrease [treatment*sex: F(1,18) = 5.180, *p* < 0.05, *p* < 0.05 *vs* Veh females]. Moreover, Poly I:C upregulated TMEM119 in males only [treatment*sex: F(1,17) = 7.614, *p* < 0.05, *p* < 0.01 *vs* Poly I:C females and Veh males]. In cerebellum, the sexual dimorphism of basal gene expression was less pronounced than in the hippocampus, as only GFAP was higher, and iNOS lower, in females compared to males [main effect of sex: F(1,19) = 15.59, *p* < 0.001 for GFAP, and F(1,19) = 4.964, *p* < 0.05 for iNOS].

### MIA triggers significant changes in gut microbiota of offspring of both sexes

**Fig. 4:**
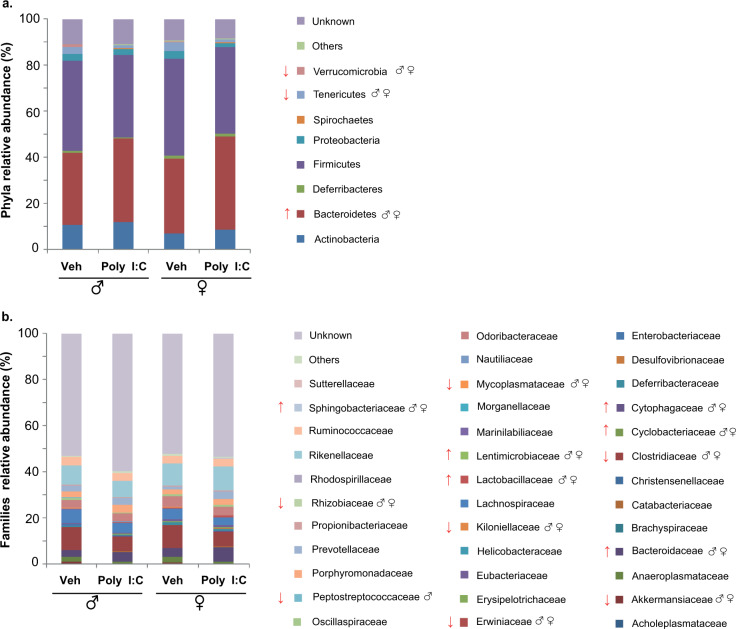
Microbiota changes induced by MIA in male and female offspring at pnd 28. Mean relative abundance (%) of gut bacteria at phyla (**a**) and families (**b**) levels; ↑ significant increase, ↓ significant decrease. Two-way ANOVA with post-hoc Tukey’s test.

*Firmicutes* and *Bacteroidetes* were the two dominant phyla in the gut microbial community of both Veh and Poly I:C offspring. At pnd 28, prenatal Poly I:C significantly increased the proportion of *Bacteroidetes* [main effect of treatment: F(1,12) = 6.098, *p* < 0.05] and appeared to decrease the proportion of *Firmicutes* (without reaching statistical significance) in both male and female offspring (Fig. 4a). Thus, the *Bacteroidetes*/*Firmicutes* ratio was higher in Poly I:C offspring compared to Veh [main effect of treatment: F(1,12) = 5.197, *p* < 0.05]. In addition, we observed a significant decrease of *Verrucomicrobia* [main effect of treatment: F(1,12) = 10.143, *p* < 0.01] and *Tenericutes* [main effect of treatment: F(1,12) = 4.459, *p* < 0.05] in Poly I:C offspring of both sexes as compared to Veh.

Relative abundances of the following families were significantly higher in Poly I:C than in Veh offspring, regardless of sex: *Bacteroidaceae* F(1,12) = 14.691, *Cyclobacteriaceae* F(1,12) = 46.394, *Cytophagaceae* F(1,12) = 31.867, *Lactobacillaceae* F(1,12) = 8.459, *Lentimicrobiaceae* F(1,12) = 27.181, all ps < 0.01; *Sphingobacteriaceae* F(1,12) = 5.572, *p* < 0.05.

By contrast, the following families were significantly less abundant in Poly I:C mice: *Akkermansiaceae* F(1,12) = 10.147, *p* < 0.01; *Clostridiaceae* F(1,12) = 6.330, *p* < 0.05; *Erwiniaceae* F(1,12) = 8.397, *p* < 0.05; *Kiloniellaceae* F(1,12) = 6.741, *p* < 0.05; *Mycoplasmataceae* F(1,12) = 10.985, *p* < 0.01, and *Rhizobiaceae* F(1,12) = 4.360, *p* < 0.05. In addition, Poly I:C males tended to show a decreased relative abundance of *Peptostreptococcaceae* compared to Veh males [treatment*sex: F(1,12) = 3.124, *p* = 0.10, just missing statistical significance after post-hoc comparisons].

**Fig. 5:**
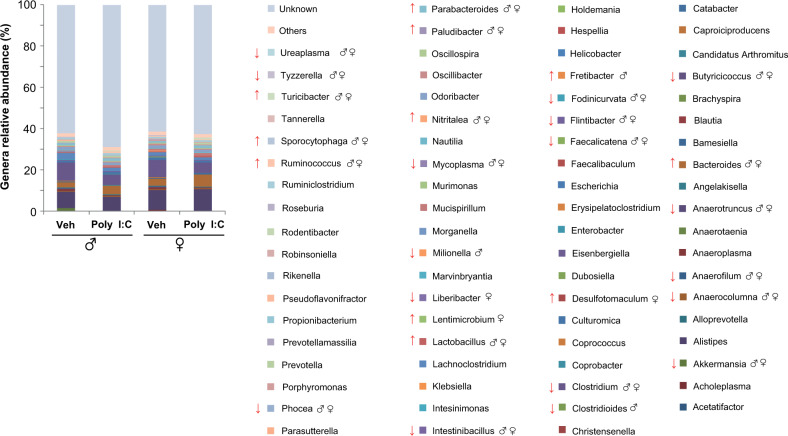
Microbiota changes induced by MIA in male and female offspring at pnd 28. Mean relative abundance (%) of gut bacteria at genera level; ↑ significant increase, ↓ significant decrease. Two-way ANOVA with post-hoc Tukey’s test.

As shown in Fig. 5, Poly I:C offspring of both sexes exhibited a significant increase of the following genera: *Bacteroides* F(1,12) = 16.424, *p* < 0.01; *Lactobacillus* F(1,12) = 8.305, *p* < 0.01; *Nitritalea* F(1,12) = 65.897, *p* < 0.01*; Paludibacter* F(1,12) = 8.402, *p* < 0.01; *Parabacteroides* F(1,12) = 6.812, *p* < 0.05; *Ruminococcus* F(1,12) = 100.558, *p* < 0.01*; Sporocytophaga* F(1,12) = 40.825, *p* < 0.01, and *Turicibacter* F(1,12) = 12.677, *p* < 0.01.

At the same time point, significant increases in relative abundance of some genera were detected in one of the two sexes only: *Desulfotomaculum* [treatment*sex: F(1,12) = 9.243, *p* = 0.01, *p* < 0.05 Poly I:C females *vs* Veh females], *Fretibacter* [treatment*sex: F(1,12) = 4.961, *p* = 0.04, *p* < 0.01 Poly I:C males *vs* Veh males]*, Lentimicrobium* [treatment*sex: F(1,12) = 3.317, *p* = 0.09, *p* < 0.01 Poly I:C females *vs* Veh females].

Poly I:C offspring, regardless of sex, showed also a significant decrease of the following genera: *Akkermansia* F(1,12) = 0.132, *p* < 0.01; *Anaerofilum* F(1,12) = 50.517, *p* < 0.01; *Anaerotruncus* F(1,12) = 7.811, *p* < 0.01; *Anaerocolumna* F(1,12) = 6.098, *p* < 0.05; *Butyricicoccus* F(1,12) = 11.074, *p* < 0.01; *Clostridium* F(1,12) = 7.402, *p* < 0.05; *Faecalicatena* F(1,12) = 6.165, *p* < 0.05; *Fodinicurvata* F(1,12) = 6.328, *p* < 0.05; *Flintibacter* F(1,12) = 13.703, *p* < 0.01; *Phocea* F(1,12) = 12.477, *p* < 0.01, *Intestinibacillus* F(1,12) = 7.662, *p* < 0.05; *Mycoplasma* F(1,12) = 11.491, *p* < 0.01; *Tyzzerella* F(1,12) = 22.715, *p* < 0.01, and *Ureaplasma* F(1,12) = 10.973, *p* < 0.01. A significant decrease of *Liberibacter* were observed in Poly I:C females only [treatment*sex: F(1,12) = 3.502, *p* = 0.08, *p* < 0.05 *vs* Veh females].

At pnd 120, most of the differences observed between Poly I:C and Veh offspring at pnd 28 were no longer detectable. At the phylum level, similarly to pnd 28, the dominant composition of the gut microbiota was *Firmicutes* and *Bacteroidetes*, but no statistical significant differences were observed between the phyla in both sexes (Supplementary Fig. 1a).

The only changes persisting at pnd 120 were those relative to the family *Mycoplasmataceae* as well as the genera *Ureaplasma* and *Mycoplasma*. In Poly I:C males and females, the *Mycoplasmataceae* were found down-represented as compared to Veh mice [main effect of treatment: F(1,12) = 20.218, *p* < 0.01], Supplementary Fig. 1b. At the genus level, Poly I:C offspring of both sexes revealed a significant decrease in the relative abundance of *Ureaplasma* [main effect of treatment: F(1,12) = 14.358, *p* < 0.01], and *Mycoplasma* [main effect of treatment: F(1,12) = 20.058, *p* < 0.01] together with the reduction of *Alloprevotella* [main effect of treatment: F(1,12) = 5.286, *p* < 0.05] and *Parasutterella* [main effect of treatment: F(1,12) = 6.575, *p* < 0.05] as compared to Veh mice, Supplementary Fig. 2.

### Correlational analyses concerning gut microbiota, neuroinflammatory and behavioral data

Since siblings (derived from the same mother/litter) share the same maternal and home-cage microbiota at weaning (pnd 28) [31, 32], we correlate gut microbiota weaning data with either behavioral items or neuroinflammatory markers assessed in littermates. Moreover, because Poly I:C-induced behavioral deficits were evident in both sexes, we focused on those bacterial taxa and neuroinflammatory markers found comparably altered in Poly I:C male and female mice at pnd 28. The major result of correlational analyses between gut microbiota and behavioral phenotype concerned the abundance of *Bacteroidetes* phylum (found significantly increased in Poly I:C mice) negatively associated with the percentage of PPI (found significantly lower in Poly I:C mice) (*r* = −0.605, *p* < 0.05).

Significant correlations between gut microbiota and neuroinflammatory profile as well as neuroinflammatory profile and behavioral phenotype are reported in Table 1 (for the complete correlation matrices see Supplementary Tables 1, 2).

**Table 1:** Correlations of neuroinflammatory markers with bacterial taxa and behaviors.

Bacterial taxa	Neuroinflammatory markers			*r*	*p*
	Markers	Brain region	Age		
Bacteroidetes	TNF-α	Cerebellum	pnd 28	0.584	<0.05
Firmicutes				−0.616	<0.01
Tyzzerella (Firmicutes)	BDNF	Cerebellum	pnd 28	0.625	<0.01
Turicibacter (Bacillota)	BDNF	Cerebellum	pnd 28	−0.514	<0.05
Social behavior	TNF-α	Cerebellum	pnd 28	−0.535	<0.05
			pnd 120	−0.641	<0.01
	ARG-1	Hippocampus	pnd 120	0.578	<0.05
Prepulse inhibition	TNF-α	Cerebellum	pnd 28	−0.621	<0.01
			pnd 120	−0.514	<0.05

## Discussion

Here we provide evidence that a single injection of Poly I:C to pregnant female mice induces long-term effects in offspring of both sexes as for (i) abnormal perseverative behavior, deficits in social interaction and sensorimotor gating impairment; (ii) neuroinflammatory response in the hippocampus and cerebellum; (iii) gut microbiota composition.

In line with previous studies on MIA models, Poly I:C offspring exhibited increased repetitive behavior [33–37] and deficits in social behavior [34, 36–38], which represent the hallmarks of ASD. As for the most common comorbid sign anxiety, we did not observe any changes in anxiety-like behavior measured through the assessment of the time spent in the center of OF arena and open-arms of EPM, according to [34]. Additionally, Poly I:C offspring displayed decreased PPI as reported by previous studies [26, 38] and in agreement with the sensorimotor gating deficits in ASD children described in a recent meta-analysis [39]. Both sexes were comparably affected by the early immune challenge, though males seemed more impaired than females. However, this trend was not statistically significant except for perseverative behavior, more prominent in Poly I:C males.

We evidenced relevant sex-related differences in the neuroinflammatory response triggered by MIA in the hippocampus and cerebellum, two brain regions consistently implicated in ASD [29, 40–42]. Different neuroinflammatory profiles were evident in males and females depending on age and brain region considered. A prominent induction of the pro-inflammatory factors TNF-α, IL-6, and iNOS, were observed in Poly I:C females only. Consistently, sex differences in neuroinflammatory profile in the hippocampus have been documented in adult MIA offspring [43].

MIA affected also Arg-1 and BDNF expression in the hippocampus of offspring of both sexes at adolescence but these alterations were attenuated at later stages of development. Dysregulation of BDNF and its relative signaling pathway has been involved in ASD phenotype [44, 45], as well as in altered developmental pruning in multiple brain regions [46, 47]. An interesting finding concerns the regulation of ARG-1, which increased in the hippocampus of MIA offspring in adolescence but significantly decreased in adulthood; the latter is in agreement with decreased ARG-1 expression reported in the hippocampus and prefrontal cortex of offspring prenatally exposed to Poly I:C [48] or LPS [49], respectively. ARG-1 is an alternative microglial activation marker, which competes with iNOS for the substrate L-arginine and inhibits the production of nitric oxide (NO), thus participating to the resolution of inflammation [50]. Besides this role, ARG-1 plays a critical role in neurodevelopment, neurogenesis and axonal repair mechanism [51]. In a recent paper, ARG-1 microglial conditional knockout mice display impaired neuronal plasticity and cognitive deficits [52]. Interestingly, microglial ARG-1 overexpression in dentate gyrus ameliorates behavioral deficits, including PPI induced by MIA, suggesting a protective role of ARG-1 in this model [48]. In agreement, in our study, we found a positive association between ARG-1 expression in the hippocampus in adulthood and social behavior, supporting the hypothesis of ARG-1 modulation of MIA behavioral phenotype.

In the cerebellum, evidence of reactive gliosis was indicated by elevated levels of TNF-α in both sexes and of the microglial markers TREM2 and CD68, and the astrocytic marker GFAP in females at adolescence. TREM2 is a member of TREM immunoglobulin superfamily required for the acquisition of a full inflammatory profile in response to tissue damage [53]. TREM2 activation is associated to phagocytosis and synaptic pruning [54]. Therefore, TREM2 upregulation, together with that of CD68, a marker of phagolysosomal activity, may suggest sex-specific alterations in microglial phagocytosis, resulting in abnormal synaptic pruning in the female adolescent cerebellum. At adult stage, the upregulation of the above markers was no longer evident, except for TNF-α, which was elevated in both sexes. The long-lasting increase of TNF-α in the cerebellum of Poly I:C male and female offspring as well as its association with the impairment in social behavior and PPI are worth of note. Recent data showed that TNF-α inhibitor administered during adolescence and early adulthood alleviated PPI deficits in rats neonatally treated with Poly I:C [55].

Interestingly, TNF-α levels are increased in blood, CSF, and brain tissue of ASD patients [4, 8–10, 56–58]. Notably, blood concentrations of TNF-α have been positively associated with severity of ASD symptoms [57, 59]. TNF-α exerts a pivotal role in several physiological processes such as synaptic plasticity and glutamate-mediated cytotoxicity, altered in ASD [60, 61]. The increase of TNF-α in cerebellum was paralleled by an upregulation of Tmem119 in males and IL-6 in females at pnd 120. A female-specific downregulation of IL-1β was also found, pointing out the persistent sexual dimorphic response to MIA also in this brain region, which deserves further consideration. Overall, the gene expression data indicate that, in adulthood, the higher susceptibility of females to prenatal Poly I:C exposure observed at weaning was no longer detectable in the hippocampus. In contrast, a long-lasting response to Poly I:C was still evident in the cerebellum, mainly in females. Since glial cells, particularly microglial cells, are considered the main source of these inflammatory mediators in the brain, changes in neuroinflammatory parameters here observed could actually reflect alterations in microglial activation state. Our findings are consistent with evidence of gliosis in different brain regions, including cerebellum, of autistic patients [4, 5, 7, 58, 62]. Interestingly, MIA reduced the number of Purkinje cells in a sex-dependent manner in specific cerebellar regions [63]. Further studies are warranted to elucidate the possible causative link between inflammatory and structural alterations in cerebellum widely documented in ASD.

Despite the prevalence of specific microbial strains differs between ASD studies and no clear trend for a gut microbiota profile in ASD has been described so far, our analysis revealed MIA changes in specific microbial taxa (mainly at pnd 28), similar to those documented in some studies on ASD children [64]. It is worth of note that the increase of phylum *Bacteroidetes* and the decrease of phylum *Verrucomicrobia* at pnd 28 are consistent with recent evidence on ASD children [64–67]. Interestingly, in our study the proportion of phylum *Bacteroidetes* correlated negatively with percentage of PPI. In addition, it was positively associated with expression of TNF-α in cerebellum at pnd 28, confirming the critical role of gut microbiota on the regulation of neuroinflammatory response [68]. At the family level, the decreased abundance of *Peptostreptococcaceae* found in Poly I:C male mice agrees with findings in ADS children [69]. Interestingly, *Peptostreptococcaceae*, enriched in neurotypical children, were positively correlated with levels of the short-chain fatty acid butyrate, found lower in ASD children [69].

At the genus level, the significant increase of *Ruminococcus* and *Turicibacter* and decrease in *Akkermansia*, *Tyzzerella*, *Ureaplasma* and *Mycoplasma* are found in Poly I:C juveniles of both sexes, in line with studies on ASD children [64, 67, 70–73]. Notably, the increase of *Turicibacter* and decrease of *Tyzzerella* detected at pnd 28 were associated with BDNF decrease in cerebellum at the same time point. However, the conflicting nature of the results obtained on the interaction between gut microbiota and ASD [74, 75] does not warrant univocal conclusion.

It is worth of note that most of significant changes in gut microbiota following Poly I:C administration were detected at weaning (pnd 28), whereas behavioral effects persist at an age (pnd 63–120) when the differences in microbiota have largely disappeared. Specifically, microbiota composition in offspring at weaning reflects the maternal microbiota [76] and therefore, it can be assumed that perturbations of the maternal microbiota during gestation, through maternal infection, primes fetal neurodevelopment during this critical period and might affect behavioral functions of the offspring at later ages [77, 78]. The importance of early–life microbiota on behavior regulation is also confirmed by evidence demonstrating that recolonization with “normal” microbiota at weaning, but not later in life, is effective to reverse social deficits in GF mice [16].

Unfortunately, our experimental design did not allow us to correlate measures (behavior, microbiota and neuroinflammation) obtained from the same individual, representing a limitation to draw conclusions. However, the correlational analyses performed on siblings (individuals derived from the same litter) and including all animals, irrespectively of their prenatal treatment, support the association between these variables.

Notably, the association between gut microbiota and behavior in MIA model is further supported by a recent study reporting deficits in recognition memory in control mice that received gut microbiota from MIA mouse donors [79].

Overall, our results show that behavioral alterations of MIA offspring are accompanied by signs of chronic neuroinflammation as well as gut dysbiosis confirming the usefulness of this model to disentangle driving factors leading to altered behavioral phenotypes in ASD and other neuropsychiatric disorders (e.g., schizophrenia).

Importantly, our study sets the ground for further studies to verify whether sex-related differences in neuroinflammation and gut microbiota induced by MIA could extend also to behavioral domains not here investigated (i.e., the cognitive one), or might confer susceptibility to - or protect against -secondary challenges (e.g., bacterial infection).

## Supplementary information

Supplementary Figure Legends

Supplementary Figure 1. Microbiota changes induced by MIA in male and female offspring at pnd 120.

Supplementary Figure 2. Microbiota changes induced by MIA in male and female offspring at pnd 120.

Supplementary Table 1. Correlation matrix of neuroinflammatory markers (pnd 28) with bacterial taxa (pnd 28) and behaviors

Supplementary Table 2. Correlation matrix of neuroinflammatory markers (pnd 120) with behaviors
