# Supplementary material for: Maternal immune activation induces autism-like changes in behavior, neuroinflammatory profile and gut microbiota in mouse offspring of both sexes
Source: Transl Psychiatry. 2022 Sep 14;12:384. doi: 10.1038/s41398-022-02149-9 (PMC9474453; doi:10.1038/s41398-022-02149-9)
Supplement: Supplementary file 1 — Supplementary Figure Legends [file 41398_2022_2149_MOESM1_ESM.docx]

**Supplementary Figure Legends**

**Supplementary Figure 1.** **Microbiota changes induced by MIA in male and female offspring at pnd 120.**

Mean relative abundance (%) of gut bacteria at phyla (a) and families (b) levels; ↓ significant decrease.

**Supplementary Figure 2.** **Microbiota changes induced by MIA in male and female offspring at pnd 120.**

Mean relative abundance (%) of gut bacteria at genera level; ↓ significant decrease.
