# Supplementary figures and images for: Maternal immune activation induces autism-like changes in behavior, neuroinflammatory profile and gut microbiota in mouse offspring of both sexes
Source: Transl Psychiatry. 2022 Sep 14;12:384. doi: 10.1038/s41398-022-02149-9 (PMC9474453; doi:10.1038/s41398-022-02149-9)

**a.**

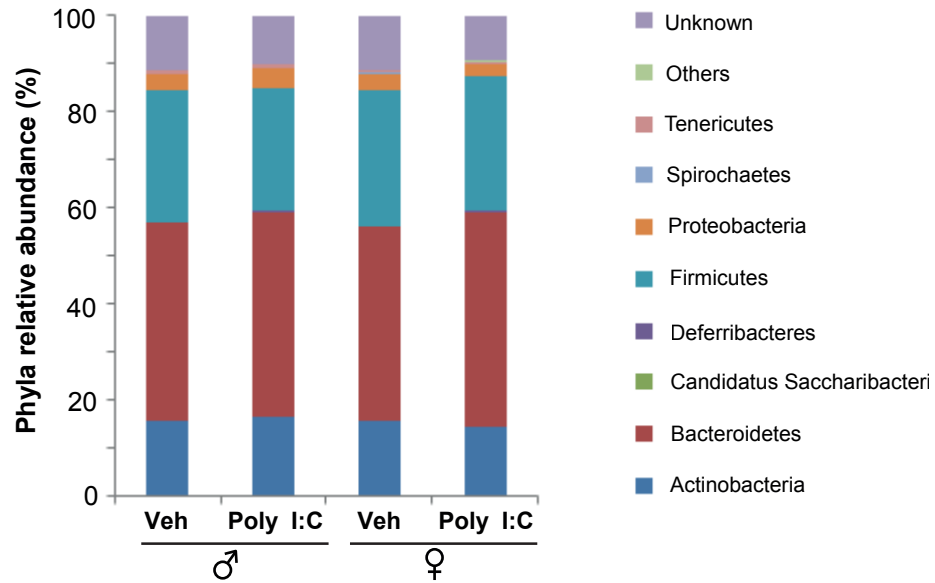

**b.**

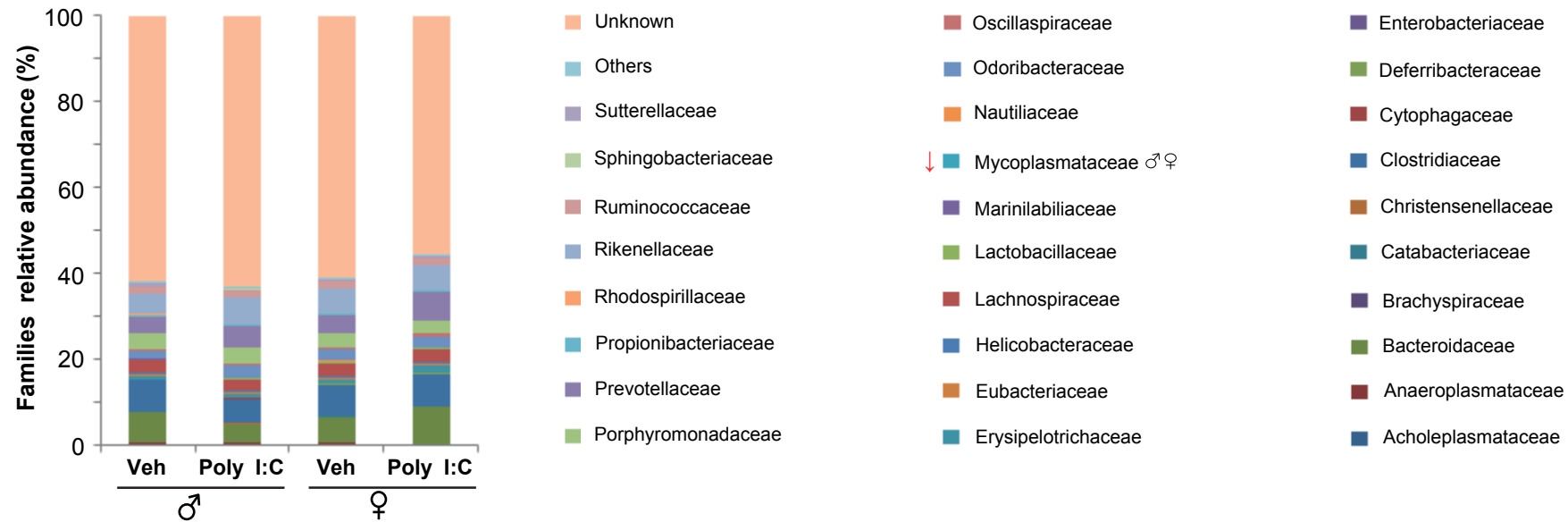

Supplement: Supplementary file 2 — Supplementary Figure 1. Microbiota changes induced by MIA in male and female offspring at pnd 120. [file 41398_2022_2149_MOESM2_ESM.pdf]

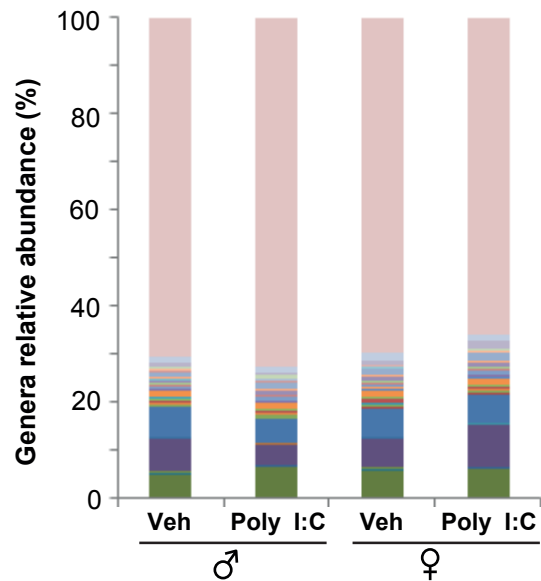

Supplement: Supplementary file 3 — Supplementary Figure 2. Microbiota changes induced by MIA in male and female offspring at pnd 120. [file 41398_2022_2149_MOESM3_ESM.pdf]
