# Supplementary material for: Maternal immune activation induces autism-like changes in behavior, neuroinflammatory profile and gut microbiota in mouse offspring of both sexes
Source: Transl Psychiatry. 2022 Sep 14;12:384. doi: 10.1038/s41398-022-02149-9 (PMC9474453; doi:10.1038/s41398-022-02149-9)
Supplement: Supplementary file 4 — Supplementary Table 1. Correlation matrix of neuroinflammatory markers (pnd 28) with bacterial taxa (pnd 28) and behaviors [file 41398_2022_2149_MOESM4_ESM.pdf]

Supplementary Table 1. Correlation matrix of neuroinflammatory markers (pnd 28) with bacterial taxa (pnd 28) and behaviors

		pnd 28									
		Bacteroidetes	Firmicutes	Tyzzerella	Turicibacter	BDNF HP	ARG1 HP	TNF-α CB	Social behavior	Spontaneous alternation	Prepulse inhibition
pnd 28	Bacteroidetes		**-.526**	-.183	.075	-.339	-.070	**.584**	-.083	-.065	**-.605**
	Firmicutes	**-.526**		-.081	.238	-.343	,063	-.616	.176	-1.732E-5	.297
	Tyzzerella	-.183	-.081		**-.692**	.625	-.028	-.032	.067	.200	-.255
	Turicibacter	.075	.238	**-.692**		-.514	.427	.080	-.379	-.402	.050
	BDNF HP	-.339	-.343	**.625**	**-.514**		-.113	-.129	.023	.046	-.027
	ARG1 HP	-.070	.063	-.028	.427	-.113		.253	-.428	-.298	-.382
	TNF-α CB	**.584**	**-.616**	-.032	.080	-.129	.253		-.535	-.301	**-.621**
	Social behavior	-,083	.176	.067	-.379	.023	-.428	**-.535**		**.796**	.161
	Spontaneous alternation	-,065	-1.732E-5	.200	-.402	.046	-.298	-.301	**.796**		.171
	Prepulse inhibition	**-.605**	.297	-.255	.050	-.027	-.382	**-.621**	.161	.171	

16 observations were used in this computation; pnd = post-natal day; HP = hippocampus; CB = cerebellum.

Values in bold are a significance level alpha < 0.05.
