# Supplementary material for: Maternal immune activation induces autism-like changes in behavior, neuroinflammatory profile and gut microbiota in mouse offspring of both sexes
Source: Transl Psychiatry. 2022 Sep 14;12:384. doi: 10.1038/s41398-022-02149-9 (PMC9474453; doi:10.1038/s41398-022-02149-9)
Supplement: Supplementary file 5 — Supplementary Table 2. Correlation matrix of neuroinflammatory markers (pnd 120) with behaviors [file 41398_2022_2149_MOESM5_ESM.pdf]

Supplementary Table 2. Correlation matrix of neuroinflammatory markers (pnd 120) with behaviors

		pnd 28					
		ARG1 HP	TNF-α CB	IL-6 CB	Social behavior	Spontaneous alternation	Prepulse inhibition
pnd 120	ARG1 HP		-.826	-.517	**.578**	.472	.283
	TNF-α CB	-.826		.582	-.641	-.493	**-.514**
	IL-6 CB	-.517	.582		-.484	-.316	-.439
	Social behavior	**.578**	-.641	-.484		.796	.161
	Spontaneous alternation	.472	-.493	-.316	.796		.171
	Prepulse inhibition	.283	**-.514**	-.439	.161	.171	

16 observations were used in this computation; pnd = post-natal day; HP = hippocampus; CB = cerebellum.

Values in bold are a significance level alpha < 0.05.
